# Gastrectasis

**Published:** 1891-08-15

**Authors:** 


### GASTRECTASIS.

Dilatation of the stomach is described in the acute and chronic form, but as the former is a rare, if not a doubtful occurrence, and has no pathological connection with the common chronic form, we shall make no reference to it at present.

Gastrectasis may arise from obstruction to the passage of its contents inwards to the duodenum, and this obstruction may be due to disease in the pylorus, such as cancer of the pylorus, cicatricial contraction following an ulcer, a chronic fibroid thickening of the submucous tissues, or a cogenital narrowing of the pyloric opening; or it may be the result of mechanical pressure on the pylorus by a growth in neighbouring organs.

Dilatation of the stomach produced by mechanical obstruction is generally associated with a considerable degree of muscular hypertrophy, just as the muscular walls of other hollow viscera, such as the bladder or the heart, become hypertrophised, and this hypertrophy in the earlier stages of the condition is probably sufficient to overcome the interference with the egress of the gastric contents. The muscular walls of the stomach, however, unlike those of the bladder or heart, have not merely to expel the contents of the viscus, but by their peculiar vennicular contractions produce a churning of the food within, the expulsion or propulsion of the partially digested food being the function more especially of the pyloric cud. Thus we find that the muscular hyertrophy is most noticeable in the neighbourhood of the pylorus, and not infrequently there is a well marked circular mass of muscular tissue around the pyloric orifice. Sooner or later, however, the frequently recurring distension of the stomach results in a permanent dilatation, and with the increasing dilatation certain pathological changes take place in the gastric mucous membrane. Thus the rugæ are effaced by constant stretching, so that the surface of the mucous membrane becomes smooth. Dr. Samuel Fenwick states that microscopically the glandular structure will be found to have suffered from the long continued stretching. In one case he found the tubes visible, but widely separated from each other, the gastric cells being large and fatty. In another case the destruction had proceeded still further; a larger proportion of the tubes had been destroyed, and were replaced by fibrous tissue, the muscular tissue being also thin and fibrous.

There is another group of cases of dilatation of the stomach, which are the result, not of mechanical obstruction, but of a paretic condition of the muscular walls. Dr. Wilks has described a case in which this followed an injury to the splanchnic nerves, and refers to a general dilatation of the stomach and intestines from injury to the solar plexus. It is to this class of gastrectasis that Dr. Wolff, of Philadelphia, has especially directed attention.* He affirms that the gastric ectasis resulting from muscular atrophy of the stomach, the atonic dilatation of the stomach, may result by muscular exhaustion, or the insufficient nourishment of the muscle consequent on anæmia, chlorosis, and nervous affections. Under this class may also be counted the chronic gastric catarrh and the loss of chemical digestive secretion which is known to form a stimulus for gastric peristalsis. The nervous elements contributing to muscular atony of the stomach are not sufficiently explained, though there seems, no doubt, a direct relation between a lack of intestinal peristalsis with gastric ectasis. Gastric dilatation is more apt to be met with in middle-aged people than in the young, though it has been reported in the young and even in children. The development of this condition is always more or less gradual, and on account of the prevailing symptoms it is in the beginning generally considered as a simple dyspepsia, attributable to errors in diet or nervous causes. To the general symptoms there is to be added as a characteristic feature of extreme dilatation, the vomiting which occurs at intervals, when by super added ingestra and the fermentative changes they are undergoing the distended stomach becomes irritated and reflex vomiting takes place. This may occur at periods of several days only. There is one unmistakable feature of the vomiting of gastrectasis, and that is the enormous quantities that are ejected at one time, the comparative ease with which the emetic act takes place, and the character of the ejecta, which are composed of food particles of several of the meals previously taken, together with the fermented appearance it presents and the gases arising therefrom on standing. Dr. Wolff went on to say that the chemistry of the functional dilatation is, in cases where marked chronic catarrhal symptoms are absent, a normal one, or rather, perhaps, one of subacidity; in complete dilatation from pyloric stenosis, it is held by such eminent workers as Ewald and Riegel that in all cases non-cancerous they had found hydro-chloric acid, though in a diminished amount. He has not been able to confirm their statements, and demonstrated a case in which the gastrectasis resulted from complete pyloric stenosis, and which during the whole period of treatment was accompanied by anacidity, and which patient, though apparently cured of his stenosis, and improved in dilatation, presented the picture of total anacidity. Another case was shown of a functional dilatation, with chronic gastric catarrh, which at the commencement of treatment had total anacidity, but in whom acidity was returning gradually as the result of treatment.

* *Therap. Gaz.*, July, 1891.

